# Rapid Myopic Progression in Childhood Is Associated With Teenage High Myopia

**DOI:** 10.1167/iovs.62.4.17

**Published:** 2021-04-14

**Authors:** Carla Lanca, Li-Lian Foo, Marcus Ang, Chuen-Seng Tan, Biten Kathrani, Hla Myint Htoon, Donald Tan, Quan V. Hoang, Noel Brennan, Seang-Mei Saw, Charumathi Sabanayagam

**Affiliations:** 1 Singapore Eye Research Institute, Singapore; 2 Ophthalmology and Visual Science Academic Clinical Program, Duke-NUS Medical School, Singapore; 3 Singapore National Eye Centre, Singapore; 4 Saw Swee Hock School of Public Health, National University of Singapore and National University Health System, Singapore; 5 Johnson & Johnson Vision, Johnson & Johnson Vision Care, Singapore; 6 Johnson & Johnson Vision, Johnson & Johnson Vision Care, Jacksonville, FL, United States; 7 Yong Loo Lin School of Medicine, National University of Singapore and National University Health System, Singapore

**Keywords:** myopia, progression, teenagers

## Abstract

**Purpose:**

The purpose of this study was to evaluate the association of childhood progression of spherical equivalent (SE) with high myopia (HM) in teenagers in the Singapore Cohort of Risk factors for Myopia (SCORM).

**Methods:**

We included 928 SCORM children followed over a mean follow-up of 6.9 ± 1.0 years from baseline (6–11 years old) until their teenage years (12–19 years old). Cycloplegic autorefraction and axial length (AL) measurements were performed yearly. The outcomes in teenagers were HM (SE ≤ −5 diopter [D)], AL ≥ 25 mm, SE and AL. Three-year SE and AL progression in childhood and baseline SE and AL with outcomes were evaluated using multivariable logistic or linear regression models, with predictive performance of risk factors assessed using the area under the curve (AUC).

**Results:**

At the last visit, 9.8% of teenagers developed HM and 22.7% developed AL ≥ 25 mm. In multivariate regression analyses, every −0.3 D/year increase in 3-year SE progression and every 0.2 mm/year increase in 3-year AL progression were associated with a −1.14 D greater teenage SE and 0.52 mm greater teenage AL (*P* values < 0.001). The AUC (95% confidence interval [CI]) of a combination of 3-year SE progression and baseline SE for teenage HM was 0.97 (95% CI = 0.95 – 0.98). The AUC of 3-year AL progression and baseline AL for teenage AL ≥ 25 mm was 0.91 (95% CI = 0.89 – 0.94).

**Conclusions:**

Three-year myopia progression in childhood combined with baseline SE or AL were good predictors of teenage HM. Clinicians may use this combination of factors to guide timing of interventions, potentially reducing the risk of HM later in life.

The increase in myopia prevalence in the last few decades impose a significant public health concern with high economic costs for the society.^1^^–^^5^ High myopia (HM, spherical equivalent [SE] ≤ −5 diopter [D] or −6 D) in young adults is reaching epidemic proportions in East and Southeast Asia with prevalence rates reported to range between 8% and 21.6% from childhood to young adulthood.^6^^–^^9^ Individuals with HM have increased susceptibility to visual impairment (VI) and blindness due to macular and retinal complications in later adulthood.^10^^–^^15^ Myopic macular degeneration (MMD) where significant retinal or optic nerve lesions are present is a common cause of irreversible VI in Asian populations.^16^^,^^17^

Previous longitudinal studies, from childhood to young adulthood, have identified childhood risk factors, including younger age of onset of myopia^18^^–^^20^ and higher myopic baseline SE,^21^ to be predictive of HM later in life. The Singapore Cohort of Risk factors for Myopia (SCORM) had shown that earlier age of onset of myopia was a risk factor for the development of HM in 11-year-olds.^18^ Similar results were found in a Denmark study, where children (9 to 12 years at baseline) with younger age of myopia onset were more likely to develop HM after 8 years of follow-up (17–20 years old).^19^ In a population-based prospective cohort study of twins in Guangzhou, China, with a 12-year annual follow-up, the risk of HM was high in children with myopia onset during the early school ages (mean ± standard deviation [SD] age at myopia onset, 11.7 ± 2.0 years).^20^ In the Correction of Myopia Evaluation Trial (COMET), including children aged 6 to 11 years at baseline, younger age and higher myopic SE at baseline were identified as significant risk factors for developing HM after 7 years of follow-up.^21^

Better understanding of the impact of early childhood myopia progression on the development of HM later in life (with the associated risk of VI) can potentially guide clinical management for timely interventions. However, few, if any, prospective longitudinal studies have examined the specific role of early childhood myopia progression as a risk factor for teenage HM. In the current study, we aimed to evaluate the association of short- and long-term SE progression in childhood with teenage HM using data from the longitudinal SCORM cohort.

## Methods

### Study Population

**Figure 1.**
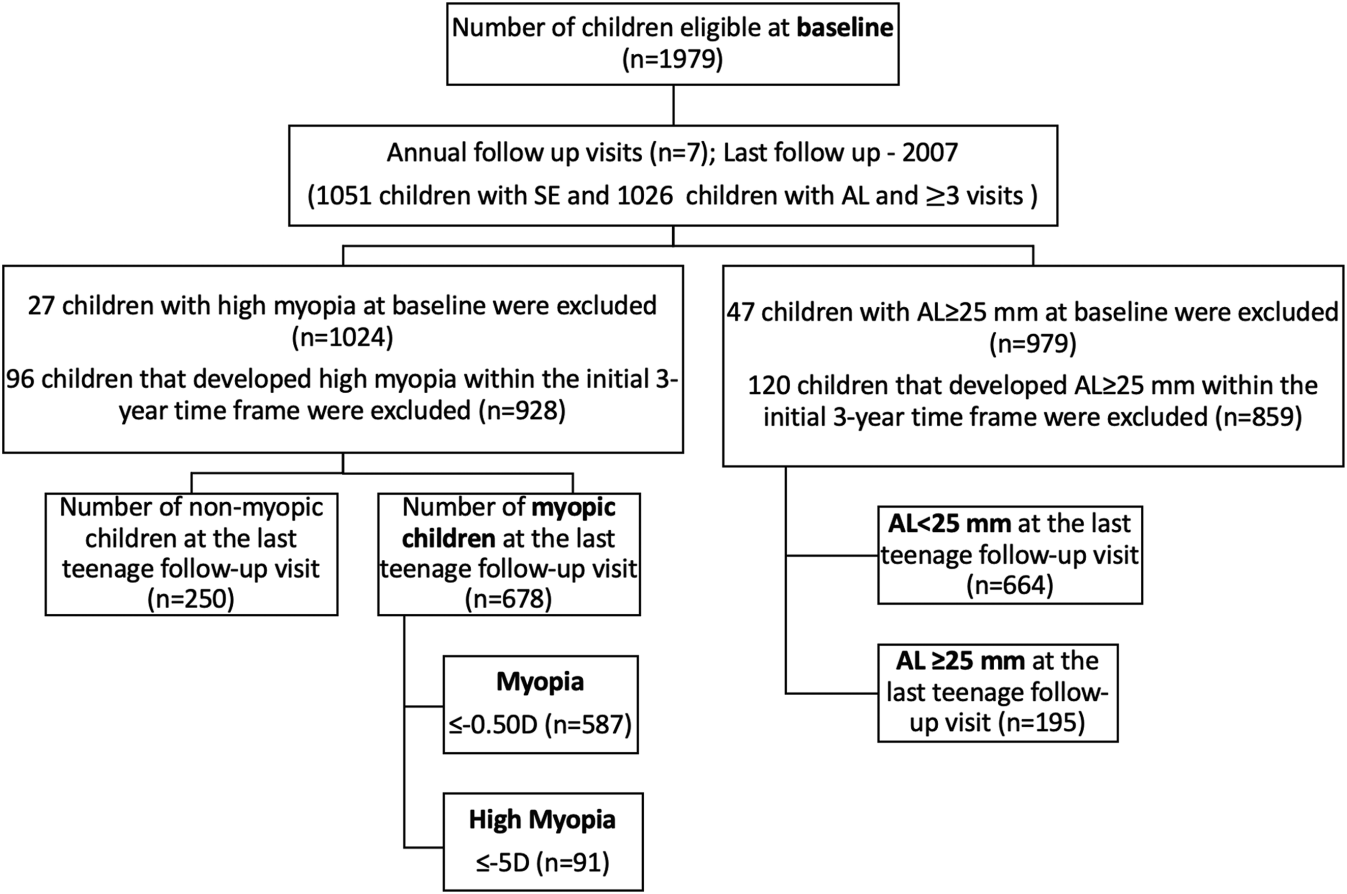
Follow-up status of eligible children for teenage years (12–19 years old) in SCORM.

SCORM is a prospective cohort whereby children from grades 1 to 3 were recruited from three Singapore schools (*n* = 1979), and the methodology has been described previously.^22^^–^^24^ At baseline, children were excluded if they had serious medical conditions, such as leukemia or heart disorders, or syndrome-associated with myopia, or any eye disorders, such as congenital cataract.^25^^–^^27^ Data for this study was derived from 1051 children who attended a minimum of 3 visits, including the baseline (1999–2001, aged 6–11 years) and the last follow-up visit (2007, teenagers aged 12 to 19 years, consistent with the World Health Organization's definition of adolescence^28^). Of the 1051 children, we excluded those with HM (*n* = 27) and AL ≥ 25 mm (*n* = 47) at baseline. We further excluded 96 children that developed HM and 120 that developed AL ≥ 25 mm within the initial 3-year time frame. For the final analysis, 928 children with SE data and 859 children with AL data were included. Seven annual follow-up visits (mean ± SD = 6.9 ± 1.0 follow-up years, range = 6 to 8) were conducted in the schools and all children examined at the last follow-up were included in this study (Fig. 1). The study was conducted in accordance with the tenets of the Declaration of Helsinki and was approved by the Ethics Committee at the Singapore Eye Research Institute and the Centralized Institutional Review Boards of the Singapore Health Services (2016/2215). Written informed consent was obtained from the parents after the nature of the study was explained.

### Eye Measurements

Cycloplegic refraction was performed yearly. After the instillation of one drop of 0.5% proparacaine, cycloplegia was achieved with 3 drops of 1% cyclopentolate instilled at 5-minute intervals. After an interval of at least 30 minutes after the last eye drop, cycloplegic autorefraction was performed with a table-mounted autorefractor (model RK5; Canon, Japan). Five measurements were performed per eye (all readings <0.25 D apart) and total mean was used for analysis. AL measurements were obtained after instillation of 1 drop of 0.5% proparacaine. Contact ultrasound biometry was performed (Echoscan model US-800, probe frequency 10 mHz; Nidek Co., Ltd., Tokyo Japan). The average of six measurements was taken and accepted only if the SD of these readings was less than 0.12 mm. SE for each eye was calculated as sphere power plus half cylinder power.

### Assessment of Covariates

Height (cm) was measured with students standing and without shoes.^29^ Questionnaires in English, Chinese, and Malay were administered to parents by a trained interviewer at the baseline visit. The questionnaires were administered to obtain demographic data, including the child's race, books read per week, and time spent outdoors (hours per week [h/wk]), as well as parental risk factors, such as mother's education level and number of parents with myopia.^23^ Parents were considered myopic if they were wearing corrective lenses to see at distance. Time spent outdoors in teenage years was recorded separately for school weekdays and school weekends using an adapted version of the Sydney Myopia Study questionnaire and was defined as the sum of outdoor leisure and outdoor sporting activities.^30^

### Predictors of Teenage HM and AL ≥ 25 mm

Myopia was defined as an SE ≤ −0.5 D and HM as an SE ≤ −5 D. The outcomes of interest were teenage HM and AL ≥ 25 mm (top tertile cut point), teenage SE and AL at the last follow-up. Baseline SE (D) and AL (mm), age of myopia onset (y, years), 3 and 1-year mean SE progression (D/y), and 3 and 1-year mean AL progression (mm/y) were evaluated as predictive factors. We measured SE progression over the initial period of 3 years in children without myopia (SE > −0.5 D) and in children with mild to moderate myopia (SE ≤ −0.5 D to >−5 D) at baseline. Three-year mean SE and AL progression, defined as annual progression over a period of 3 years, were calculated as (SE at visit 4-SE at baseline)/3 and (AL at visit 4-AL at baseline)/3. One-year mean SE and AL progression, defined as progression over a period of 1 year, were calculated as (SE at visit 2-SE at baseline) and (AL at visit 2-AL at baseline).

At baseline, age of onset of myopia was determined as the age at which corrective lenses were first prescribed to correct myopia.^18^ For children who developed myopia after the baseline visit, the age of onset of myopia was determined via annual eye examinations from the subsequent follow-up visits.

### Statistical Methods

Statistical analyses were performed on 928 children with available SE data and 859 children with available AL data. The more myopic eye (worse eye) of each subject at the last follow-up was chosen for analysis. Predictive factors, including initial 3 and 1-year SE or AL progression, SE, or AL at baseline, were analyzed as continuous variables (per SD increase). We examined the association of predictive factors with teenage HM and AL ≥ 25 mm using multiple logistic regression models and teenage SE and AL using multiple linear regression models, adjusting for confounders, such as age at baseline, height (only for AL), gender, race, mother's education, parental myopia, outdoor time, books read per week, and length of follow-up, with manual backward stepwise approaches. We performed two distinct analyses (1) in children with SE measures at the last follow-up (*n* = 928) and (2) in children with AL measures at the last follow-up (*n* = 859). For analysis in children with myopia at the last follow-up, we additionally evaluated age of onset of myopia.

Receiver operating characteristics (ROCs) and areas under the curve (AUC) curves associated with logistic regression models were used to assess their operating characteristics for HM and AL ≥ 25 mm. Risk factors were progressively added comparing the discriminative ability using AUC to obtain the best clinical model. To correct the optimism bias, we performed a 10-fold cross validation for the AUC by averaging it across each fold and bootstrapping the cross-validated AUC to obtain its confidence intervals (CIs). We have also computed the estimated probability (or risk) of HM using the formula:

$$\mathrm{Pr}\left(HM|x_{1},x_{2}\right)=\frac{\exp\left\{\beta_{0}+\beta_{1}x_{1}+\beta_{2}x_{2}\right\}}{1+\exp\left\{\beta_{0}+\beta_{1}x_{1}+\beta_{2}x_{2}\right\}}$$

where β_0_ is the intercept term, and β_1_ and β_2_ are the log-odds of the two risk factors (i.e., *x*_1_ and *x*_2_). We plotted three estimated risks of HM as a function of one of the risk factors by specifying the other risk factor to take the value of its 25th, 50th, or 75th percentile.

Any *P* ≤ 0.05 was considered statistically significant. All statistical analyses were carried out with SPSS (IBM, Armonk, NY, USA, version 26) and Stata (Stata Statistical Software, release 11; StataCorp LP, College Station, TX, USA).

## Results

A comparison of children included in this study (*n* = 928) with those who were excluded (*n* = 1051) showed no significant difference in terms of gender. Children who were included were younger at baseline, had shorter baseline AL, less myopic baseline SE, lower proportion of Chinese race, and non-myopic parents, and had higher proportion mothers with lower education level (all *P* < 0.05; data not shown).

The characteristics of the study subjects are shown in Table 1. Of the 928 children with SE measures at the last follow-up, 9.8% (*n* = 91) developed HM, whereas 73.1% (*n* = 678) had myopia. Of the 859 children with AL measures, 22.7% (*n* = 195) of the children developed teenage AL ≥ 25 mm. Of those with both measures (*n* = 823), 4.4% (*n* = 36) developed both HM and teenage AL ≥ 25 mm. During the study follow-up, of the non-myopes at baseline (*n* = 680), 61.2% (*n* = 416) developed mild to moderate myopia, and 2.4% (*n* = 16) developed teenage HM; of the mild to moderate myopes at baseline (*n* = 248) 30.2% (*n* = 75) developed teenage HM.

**Table 1.** Demographics and Characteristics of Children Included in the Study

	n	Mean (SD) or (%)
Age at baseline (y)	928	7.8 ± 0.8
Age of onset of myopia (y)	677	9.4 ± 2.2
Gender (%)		
Boys	450	48.5
Girls	478	51.5
Race (%)		
Chinese	645	69.5
Malay	207	22.3
Indian and others	76	8.2
Height (cm)	925	126.4 ± 7.7
Length of follow-up (y)	928	6.9 ± 1.0
Mother's education (%)		
Secondary school or less	331	35.7
Above secondary school	597	64.3
Outdoor time (h/wk)	914	3.25 ± 1.99
Books read per week (*n*)	928	2.41 ± 2.42
Parental myopia (%)		
None	414	44.6
One	359	38.7
Both	155	16.7
Baseline SE (D)	928	0.002 ± 1.09
Baseline AL (mm)	914	23.14 ± 0.79
3-year SE progression (D/y)	885	−0.43 ± 0.33
1-year SE progression (D/y)	906	−0.48 ± 0.43
3-year AL progression (mm/y)	873	0.21 ± 0.17
1-year AL progression (mm/y)	881	0.25 ± 0.35

y, years; h/wk, hours per week; D, diopetr; n, number; SD, standard deviation.

For some variables “*n*” may not add up to 928 due to missing data.

**Figure 2.**
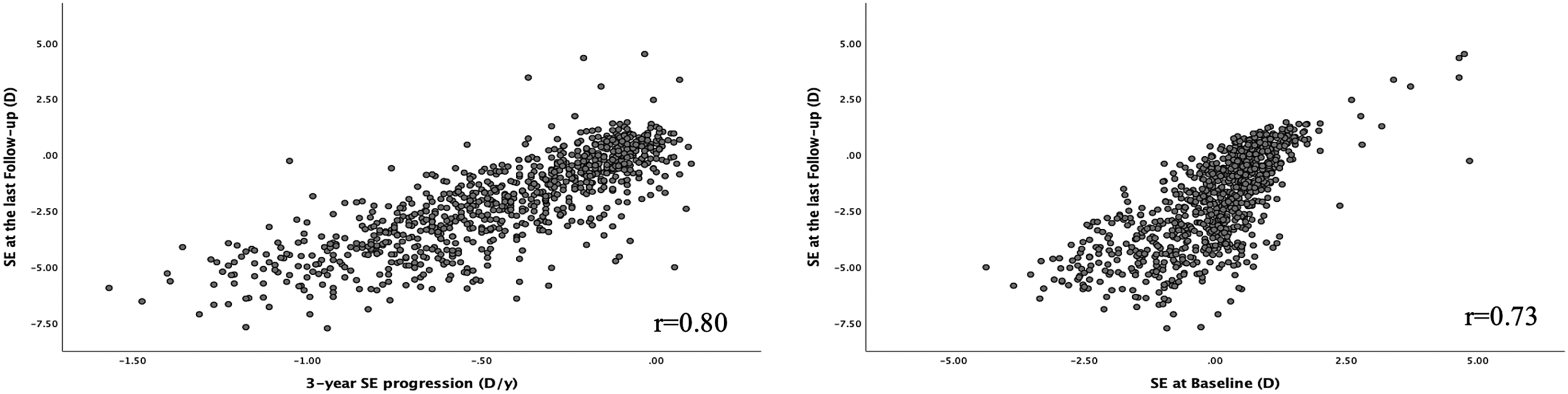
**Panel of scatter plots of teenage SE against SE at baseline and 3-year SE progression in SCORM.** Three-year SE progression (D/y) was calculated as ((SE at visit 4 – SE at baseline)/3).

Among all children, the mean SE (± SD) of the more myopic eye was −2.03 ± 2.04 D at the last follow-up visit, whereas the mean AL was 24.38 ± 1.05 mm at the last follow-up visit. Mean SE progressed by −0.48 ± 0.43 D/y in the first year of the study and by −0.43 ± 0.33 D/y in the first 3 years of the study (from 7.8 ± 0.8 years old at baseline to 10.8 ± 0.8 years old at visit 4). Figure 2 shows that the teenage SE was highly correlated with the 3-year SE progression (*r* = 0.80) and SE at baseline (*r* = 0.73). The correlation between teenage SE and 1-year SE progression was lower (*r* = 0.66; data not shown).

Of the 678 children with myopia at the last follow-up, 86.6% had mild to moderate myopia. The mean age of myopia onset was 9.4 ± 2.2 years with 41.1% developing myopia at or after the age of 10 years. Of the 91 children with HM, the mean age of myopia onset was 7.5 ± 1.1 years and mean age of HM onset was 13.3 ± 1.5 years. On average, children with myopia had 5.2 ± 2.3 years of myopic life (myopia duration in years) at the last follow-up and SE progressed by −0.59 ± 0.42 D/y in the first year of the study and by −0.55 ± 0.30 D/y in the first 3 years of the study. Higher duration of myopic life was correlated with more myopic teenage SE (*r* = −0.57). Eighteen percent of the children had progressive myopia (3-year mean SE progression ≤ −0.75 D/y). Sixty-seven percent of the children with progressive myopia developed HM at the last-follow-up, compared with only 33% in the nonprogressive group (*P* < 0.001; data not shown).

Table 2 shows the baseline characteristics of children stratified by teenage HM and AL ≥ 25 mm. Children with teenage HM and AL ≥ 25 mm developed myopia at a younger age and had significantly lower outdoor time compared with children without teenage HM or without AL ≥ 25 mm. These children were also more likely to be Chinese, have mothers with above secondary school education, and with both parents more likely to be myopes. Their baseline SE and AL as well as their 3 and 1-year SE and AL progression were higher compared to those without. Additionally, children with teenage AL ≥ 25 mm were more likely to be boys and taller in height, compared to those with teenage AL < 25 mm.

**Table 2.** Comparison of Characteristics in Children With Teenage High Myopia (HM) or AL ≥ 25 mm and Children Without Teenage HM or AL < 25 mm

	Teenage High Myopia (≤−5 D; *n* = 928)^*^					Teenage AL ≥ 25 mm (*n* = 859)^*^				
	*n*	No	*n*	Yes	*P* Value	*n*	No	*n*	Yes	*P* Value
Age at baseline (y)	837	7.8 ± 0.8	91	7.7 ± 0.8	0.17	664	7.8 ± 0.8	195	7.8 ± 0.9	0.69
Age of onset of myopia (y)	586	9.7 ± 2.2	91	7.5 ± 1.1	<0.001	420	10.0 ± 2.3	190	8.5 ± 1.8	<0.001
Gender (%)										
Boys	405	9082.07	45	10.0	0.85	275	72.0	107	28.0	0.001
Girls	432	90.4	46	9.6		389	81.6	88	18.4	
Race (%)										
Chinese	566	87.8	79	12.2	0.001	437	74.8	147	25.2	0.036
Malay	199	96.1	8	3.9		166	83.4	33	16.6	
Indian and others	72	94.7	4	5.3		61	80.3	15	19.7	
Height (cm)	834	126.6 ± 7.8	91	125.0 ± 7.1	0.07	662	126.0 ± 7.7	194	126.4 ± 7.8	0.51
Length of follow-up (y)	837	6.9 ± 1.0	91	6.8 ± 1.0	0.45	664	6.9 ± 1.0	195	6.8 ± 1.0	0.28
Mother's education (%)										
Secondary school or less	310	93.7	21	6.3	0.008	265	83.6	52	16.4	<0.001
Above secondary school	527	88.3	70	11.7		399	73.6	143	26.4	
Outdoor time (h/wk)	824	3.35 ± 2.02	90	2.41 ± 1.43	<0.001	653	3.35 ± 2.02	192	2.94 ± 1.93	0.012
Books read per week (*n*)	837	2.42 ± 2.48	91	2.35 ± 1.85	0.79	664	2.4 ± 2.5	195	2.5 ± 2.2	0.81
Parental myopia (%)										
None	391	94.4	23	5.6	<0.001	329	83.1	67	16.9	<0.001
One	319	88.9	40	11.1		243	74.5	83	25.5	
Both	127	81.9	28	18.1		92	67.2	45	32.8	
Baseline SE (D)	837	0.16 ± 0.97	91	−1.46 ± 1.06	<0.001	664	0.27 ± 1.06	195	−0.82 ± 1.25	<0.001
Baseline AL (mm)	824	23.07 ± 0.77	90	23.70 ± 0.79	<0.001	653	22.83 ± 0.63	193	23.66 ± 0.53	<0.001
3-year SE progression (D/y)	799	−0.38 ± 0.29	86	−0.88 ± 0.30	<0.001	639	−0.35 ± 0.30	175	−0.68 ± 0.34	<0.001
1-year SE progression (D/y)	817	−0.42 ± 0.38	89	−1.03 ± 0.44	<0.001	649	−0.38 ± 0.38	188	−0.78 ± 0.50	<0.001
3-year AL progression (mm/y)	788	0.20 ± 0.16	85	0.38 ± 0.14	<0.001	630	0.18 ± 0.16	173	0.28 ± 0.16	<0.001
1-year AL progression (mm/y)	794	0.23 ± 0.34	87	0.44 ± 0.41	<0.001	632	0.21 ± 0.34	182	0.38 ± 0.40	<0.001

y, years; h/wk, hours per week; D, diopter; n, number; SD, Standard deviation.

Mean ± SD for continuous variables and percentages for categorical variables.

* For individual variables “*n*” may not add up to 928 or 859 due to missing data.

*P* values indicate difference in participant characteristics by outcome status.

Baseline SE, 3-year SE progression and age of myopia onset were significantly associated with teenage HM (Table 3). The odds ratio (OR) was 11.43 per each SD (−0.3 D/y) increase in annual progression over a period of 3 years. Similarly, baseline AL, 3-year AL progression, and age of myopia onset were significantly associated with teenage AL ≥ 25 mm (Table 3). The OR (95% CI) for teenage HM was 2.40 (95% CI = 1.89 to 3.05) per each SD (−0.3 D/y) increase in SE progression over a period of 1 year and for teenage AL ≥ 25 mm was 1.83 (95% CI = 1.56 to 2.14) per each SD (0.2 mm/y) increase in AL progression over a period of 1 year (data not shown).

**Table 3.** Association of Key Risk Factors Alone and in Combination With Teenage High Myopia and AL ≥ 25 mm

Teenage High Myopia (≤−5 D)			Teenage AL ≥ 25 mm		
Variables	Unadjusted OR (95% CI)	Multivariable OR (95% CI)	Variables	Unadjusted OR (95% CI)	Multivariable OR (95% CI)
3-year SE progression, per SD (−0.3 D/year) ↑	4.37 (3.33-5.74)	11.43 (6.39-20.45)^*^	3-year AL progression, per SD (0.2 mm/year) ↑	2.55 (2.06, 3.14)	6.88 (4.54, 10.44)^†^
Baseline SE, per SD (−1 D) ↑	3.84 (3.03-4.87)	11.80 (6.74-20.67)^*^	Baseline AL, per SD (0.8 mm) ↑	11.46 (8.20, 16.03)	41.66 (22.71, 76.43)^†^
Age of myopia onset, per 1 year ↓	2.23 (1.84, 2.69)	3.06 (2.28, 4.10)^‡^	Age of myopia onset, per 1 year ↓	1.54 (1.39, 1.70)	1.52 (1.36, 1.70)^§^

CI, confidence intervals; OR, odds ratio; SD, standard deviation.

↑ Increase. ↓ Decrease.

* Models were adjusted for age at baseline, gender, race, mother's education, parental myopia, outdoor time, books read per week, and length of follow-up. In addition, predictive variables were mutually adjusted: baseline SE for models of 3-year SE progression; 3-year SE progression for models of baseline SE.

† Models were adjusted for age at baseline, height, gender, race, mother's education, parental myopia, outdoor time, books read per week, and length of follow-up. In addition, predictive variables were mutually adjusted: baseline AL for model of 3-year AL progression; 3-year AL progression for model of baseline AL.

^‡^Model was adjusted for age at baseline, gender, race, mother's education, outdoor time, books read per week, length of follow up, parental myopia, and 3-year SE progression.

§ Model was adjusted for age at baseline, height, gender, race, mother's education, outdoor time, books read per week, length of follow-up, parental myopia, and 3-year AL progression.

In linear regression analyses, 3-year SE progression, SE at baseline, and age of myopia onset were associated with teenage SE (Table 4). Three-year SE progression was similar by age at baseline groups, although slightly higher in children ≤ 7 years old (β = −1.20, 95% CI = −1.27 to −1.12) compared with 8 years old (β = −1.06, 95% CI = −1.14 to −0.99) and ≥ 9 years old (β = −1.06, 95% CI = −1.17 to −0.96; data not shown). Myopic life years (β = −0.13, 95% CI = −0.19 to −0.07) was associated with more myopic SE in teenagers (data not shown). Three-year AL progression, baseline AL, and age of myopia onset were associated with teenage AL (Table 4). Myopic life years (β = 0.02, 95% CI = 0.001 to 0.04) was associated with longer AL in teenagers (data not shown). One-year SE progression was also associated with teenage SE (β = −0.56 per each SD increase of −0.30 D/y, 95% CI = −0.62 to −0.51; data not shown) with slightly higher progression in children ≤ 7 years old (β = −0.61, 95% CI = −0.69 to −0.53) compared with 8 years old (β = −0.50, 95% CI = −0.59 to −0.40) and ≥ 9 years old (β = −0.52, 95% CI = −0.63 to −0.42; data not shown). One-year AL progression was also associated with teenage AL (β = 0.16 per each SD increase of 0.2 MM/year, 95% CI = 0.14 to 0.18; data not shown).

**Table 4.** Association of Key Risk Factors Alone and in Combination With Teenage SE and AL

Teenage SE (D)			Teenage AL (mm)		
Variables	Unadjusted β (95% CI)	Multivariable β (95% CI)	Variables	Unadjusted β (95% CI)	Multivariable^†^ β (95% CI)
3-year SE progression, per SD (−0.3 D/year) ↑	−1.51 (−1.58 to −1.44)	−1.14 (−1.18 to −1.09)^*^	3-year AL progression, per SD (0.2 mm/year) ↑	0.62 (0.54 to 0.69)	0.52 (0.49 to 0.56)^†^
Baseline SE, per SD (−1 D) ↑	−1.37 (−1.45 to −1.29)	−1.02 (−1.06 to −0.97)^*^	Baseline AL, per SD (0.8 mm) ↑	0.82 (0.78 to 0.86)	0.84 (0.80 to 0.87)^†^
Age of myopia onset, per 1 year ↓	−0.49 (−0.53 to −0.45)	−0.33 (−0.30 to −0.37)^‡^	Age of myopia onset, per 1 year ↓	0.18 (0.15 to 0.20)	0.15 (0.12 to 0.18)^§^

CI, confidence intervals; OR, odds ratio; SD, standard deviation.

↑ Increase. ↓ Decrease.

* Models were adjusted for age at baseline, gender, race, mother's education, parental myopia, outdoor time, books read per week, and length of follow-up. In addition, predictive variables were mutually adjusted: baseline SE for models of 3-year SE progression; 3-year SE progression for models of baseline SE.

† Models were adjusted for age at baseline, height, gender, race, mother's education, parental myopia, outdoor time, books read per week, and length of follow-up. In addition, predictive variables were mutually adjusted: baseline AL for model of 3-year AL progression; 3-year AL progression for model of baseline AL.

‡ Model was adjusted for age at baseline, gender, race, mother's education, outdoor time, books read per week, length of follow-up, parental myopia, and 3-year SE progression.

§ Model was adjusted for age at baseline, height, gender, race, mother's education, outdoor time, books read per week, length of follow-up, parental myopia, and 3-year AL progression.

**Figure 3.**
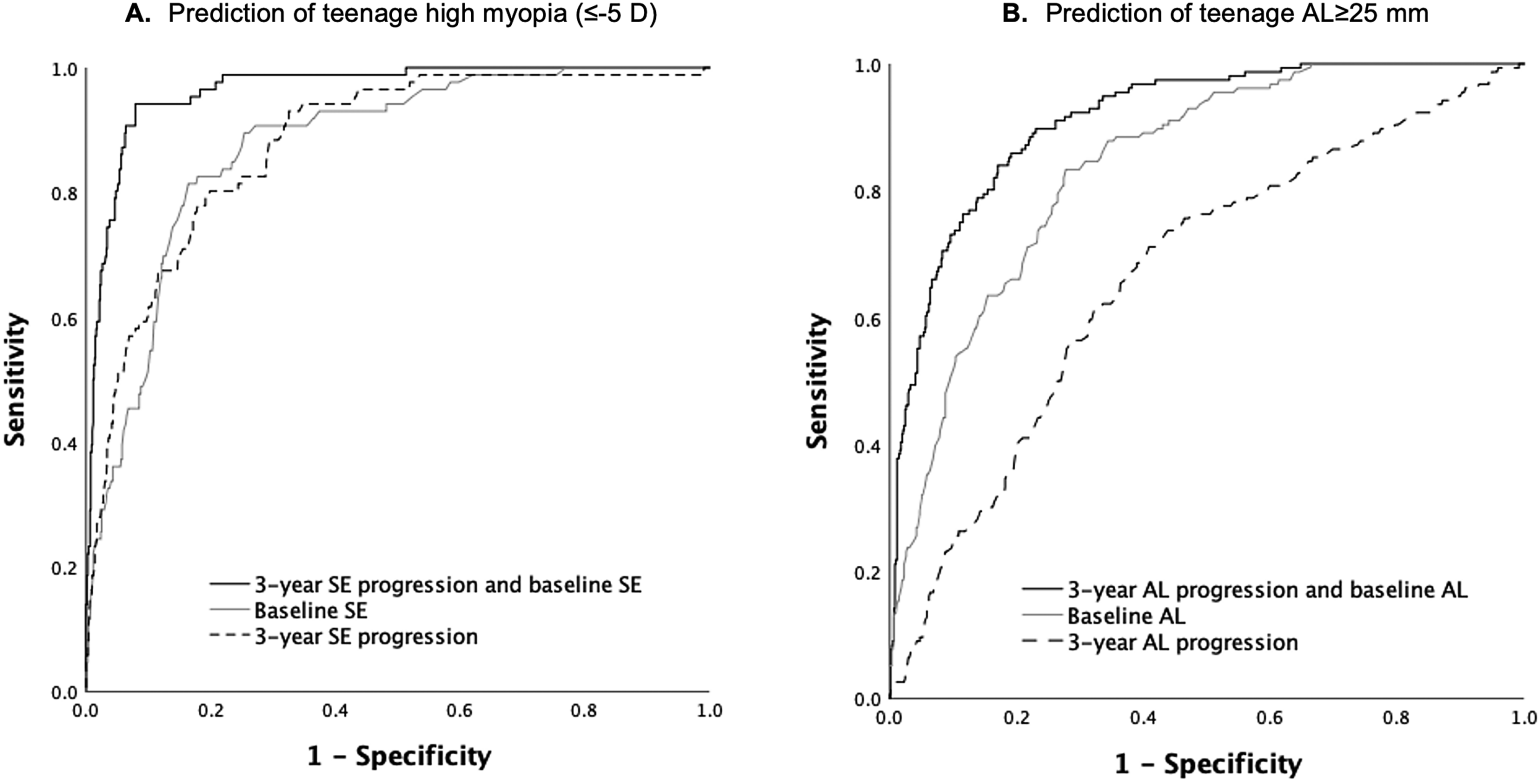
**Receiver operating curves for prediction of teenage high myopia (≤ −5 D) and AL ≥ 25 mm (top tertile cut-point; B) at the last follow-up visit. (A**) AUC_1_ = 0.97; AUC_2_ = 0.88; and AUC_3_ = 0.88. In pairwise comparisons of ROC curves, AUC_2_ was not significantly different from AUC_3_ (*P* > 0.05); (**B**) AUC_1_ = 0.91; AUC_2_ = 0.84; and AUC_3_ = 0.67. In pairwise comparisons, all ROC curves were significantly different (all *P* values < 0.05); AUC, area under the curve; AL, axial length; SE, Spherical equivalent.

The predictors of teenage HM were baseline SE with AUC (95% CI) of 0.88 (95% CI = 0.84 to 0.91), 3-year SE progression (0.88, 95% CI = 0.84 to 0.91), 1-year SE progression (0.85, 95% CI = 0.81-0.90), and age of myopia onset (0.83, 95% CI = 0.79 to 0.87). The AUC for outdoor time was 0.64 (95% CI = 0.58 to 0.69), for parental myopia was 0.63 (95% CI = 0.57 to 0.69), for books read per week was 0.50 (95% CI = 0.44 to 0.56), and for age at baseline was 0.50 (95% CI = 0.44 to 0.56). The model combining SE at baseline and 3-year SE progression together with all other risk factors, including age at baseline, gender, race, mother's education, parental myopia, outdoor time, books read per week, and length of follow-up had the highest AUC (0.97, 95% CI = 0.96 to 0.99) for predicting teenage HM (data not shown). However, a combination of the two predictors that could be applicable in clinical settings, namely 3-year SE progression and SE at baseline without other risk factors showed AUC (0.97, 95% CI = 0.95 to 0.98) similar to the model encompassing all risk factors (Fig. 3A). In sensitivity analysis, using the 10-fold cross-validation for the AUC and the bootstrapping of the cross-validated AUC we found that the mean AUC was 0.97 (bootstrap bias corrected 95% CI = 0.94 to 0.98) for a combination of 3-year SE progression and SE at baseline to predict teenage HM (data not shown). The combination of 1-year SE progression and SE at baseline showed a lower AUC of 0.94 (95% CI = 0.91 to 0.96). Other combinations, such as baseline SE with parental myopia showed lower AUC (0.89, 95% CI = 0.86 to 0.92). Age of myopia onset and 3-year SE progression combined with other risk factors were also good predictors of teenage HM (0.92, 95% CI = 0.89 to 0.95). A combination of 3-year SE progression and age of myopia onset without other risk factors, showed an AUC of 0.91 (95% CI = 0.87 to 0.94). The AUC for mild to moderate myopes at baseline (*n* = 248) to predict teenage HM was 0.91 (95% CI = 0.87 to 0.95) for a combination of 3-year SE progression and baseline SE.

For teenage AL ≥ 25 mm the best predictors were baseline AL (0.84, 95% CI = 0.81 to 0.87), 3-year AL progression (0.67, 95% CI = 0.62 to 0.71) and age of myopia onset (0.68, 95% CI = 0.62 to 0.73). The AUC for 1-year AL progression was only 0.60 (95% CI = 0.54 to 0.65). A model combining 3-year AL progression, AL at baseline and other risk factors had an AUC of 0.93 (95% CI = 0.90 to 0.95) for predicting AL ≥ 25 mm (data not shown). However, a combination of two predictors (Fig. 3B), applicable in clinical settings, 3-year AL progression and baseline AL, had AUC (0.91, 95% CI = 0.89 to 0.94) similar to the model encompassing all risk factors. In sensitivity analysis, using the 10-fold cross-validation for the AUC and the bootstrapping of the cross-validated AUC we found that the mean AUC was 0.91 (bootstrap bias corrected 95% CI = 0.89 to 0.94) for a combination of 3-year AL progression and AL at baseline to predict teenage AL ≥ 25 mm (data not shown). The combination of 1-year AL progression and AL at baseline showed slightly lower AUC of 0.89 (95% CI = 0.86 to 0.91). Other combinations, such as 3-year AL progression, age of myopia onset, and other risk factors showed lower AUC for predicting teenage AL ≥ 25 mm (0.71, 95% CI = 0.66 to 0.75; data not shown). The AUC drops further to 0.67 (95% CI = 0.62 to 0.72) for a combination of 3-year AL progression and age of myopia onset.

**Figure 4.**
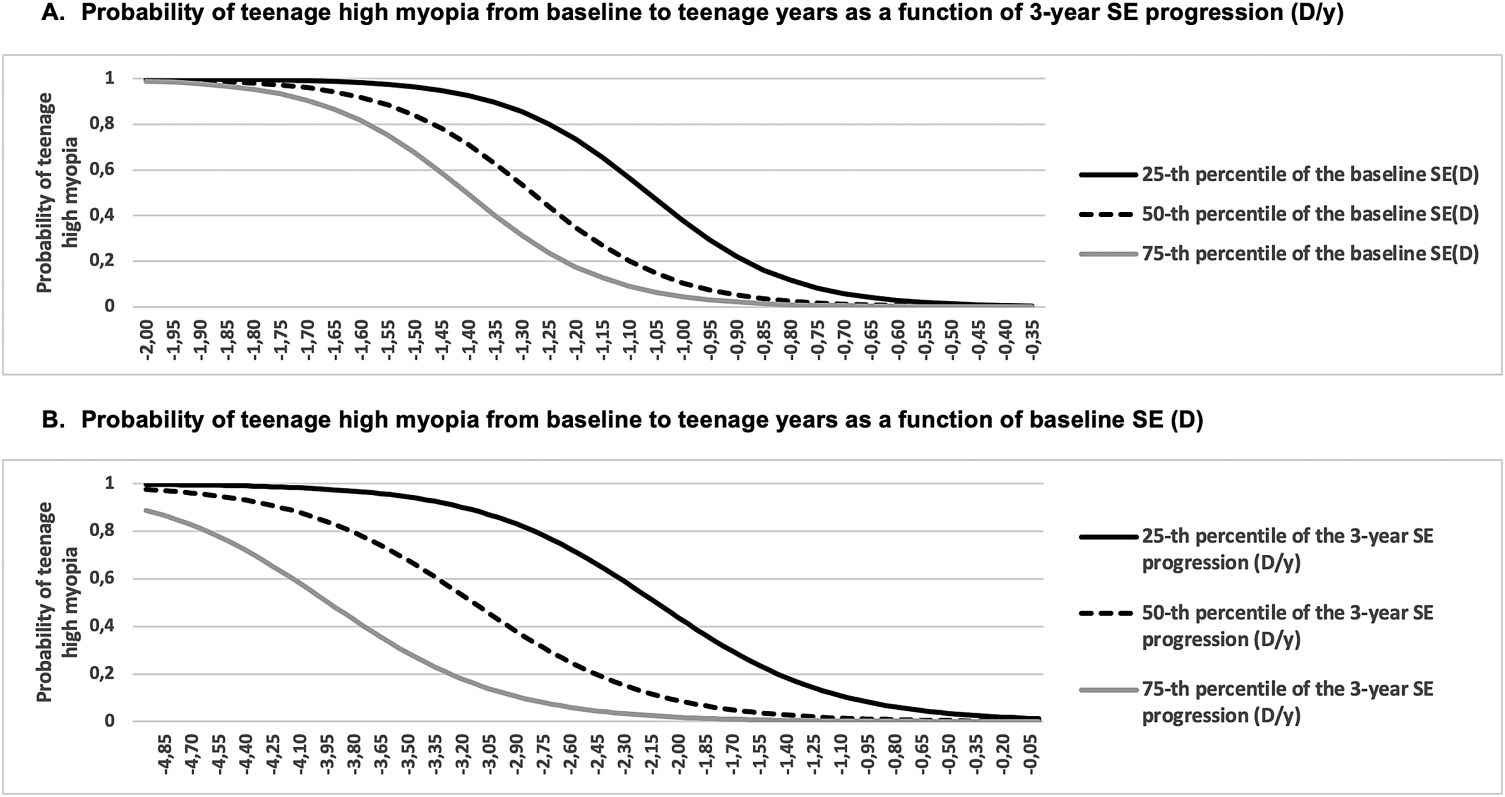
**Probability of teenage high myopia (≤ −5 D) from baseline to teenage years as a (A) function of 3-year SE progression and as a (B) function of baseline SE, using the two-factor model (i.e., 3-year SE progression and baseline SE) presented in** **Figure 3****A.** The three lines show the 25th, 50th, and 75th percentiles of the other risk factor when plotting the probability of teenage high myopia from baseline to teenage years as a function of **A** 3-year SE progression (D/y) and **B** SE at baseline (D).

Figure 4 shows the probability of teenage HM from baseline to teenage years as a (A) function of 3-year SE progression and as a (B) function of baseline SE, using the two-factor model (i.e., 3-year SE progression and baseline SE) presented in Figure 3A. The three lines show the 25th, 50th, and 75th percentiles of the other risk factor when plotting the probability of teenage HM from baseline to teenage years as a function of 3-year SE progression (D/y) and SE at baseline (D).

## Discussion

### Summary of the Results

In this longitudinal study, including children with a long follow-up, we found that 3 and 1-year childhood myopia progression (6 to 11 years old) were associated with teenage HM (12 to 19 years old). The other factors associated with teenage HM were baseline SE and age of onset of myopia. The combination of 3 or 1-year myopia progression with baseline SE or the surrogate, age of myopia onset was predictive of teenage HM. Similarly, for teenage AL ≥ 25 mm, 3 or 1-year AL progression and baseline AL emerged as top predictors. Our findings suggest the potential clinical value of utilizing information on childhood myopia and AL progression in clinical practice to devise a management plan, in terms of prevention of HM by slowing myopia progression.

### Myopia Progression and Clinical Implications

In this cohort, we observed that a rapid 3 and 1-year SE progression (−0.3 D per year) in children 6 to 11 years old led to an increased teenage mean SE in 12 to 19 years old, as compared to those with lower amounts of progression (less than −0.3 D per year), suggesting that myopia progression may be a useful indicator for the risk of developing HM later on. Although an earlier age of myopia onset is a known risk factor for the development of HM,^18^^,^^21^ few prospective studies have analyzed myopia progression as a risk factor for HM later on in life. A small retrospective study (*n* = 59) with a short follow-up showed that children aged 7 to 15 years old with HM, had greater rates of myopia progression, developed myopia earlier, and with initially higher SE levels compared with children with low myopia.^31^ In another study, a randomized clinical trial with a 22 year follow-up, including 240 school children with myopia (aged 8.8 to 12.8 years), myopia progression in childhood during the first follow-up year was identified as a risk factor associated with adulthood HM.^32^

Taken together, those findings may indicate that children with initial fast myopia progression would likely require closer monitoring and follow-up as well as timely clinical treatment interventions, because they are more likely to develop HM later in life.^21^ Thus, the results from our study may be useful to risk-stratify and guide clinical decisions in terms of myopia control management for children. For children at higher risk, more aggressive treatment, including commencing with a higher frequency or concentration of atropine eyedrops or combination treatment (e.g., atropine with multizone contact lenses), may have to be adopted for myopia control. As various myopia control interventions emerge with their own individual risk-benefits profile,^33^ our study findings could serve as an adjunctive tool for eyecare professionals to stratify children who are at greater risk of developing teenage HM.

### Myopia Progression Combined With Baseline SE or Age of Myopia Onset

Children with higher myopic progression, higher baseline myopic refractive error, and younger age of myopia onset had more severe teenage myopic refractive error. Baseline SE and age of myopia onset are known risk factors for the development of HM.^18^^,^^21^^,^^34^^,^^35^ Previous studies have attempted to establish tools to identify the risk of HM in children. Using SE centile curves, a study showed that children under lower percentiles (10th and 5th centile curves) at younger ages (mean baseline age = 12 years) were more likely to have HM at 15 years old.^34^ In the COMET, including children aged 6 to 11 years at baseline, younger children with more myopia at baseline had increased risks of developing HM after 7 years of follow-up.^21^ Our present study shows that a combination of SE progression in childhood and baseline SE myopia had good discriminative ability (97%) for teenage HM in SCORM. Using those two factors together may aid clinical decisions in myopia control treatment and counselling for children at higher risk of developing HM.

### AL Progression Combined With Baseline AL or Age of Myopia Onset

Children with higher AL progression in childhood, longer AL at baseline, and younger age of myopia onset had longer teenage AL (≥25 mm). A combination of 3 or 1-year AL progression and baseline AL were significant predictors of teenage AL ≥ 25 mm. A previous study has tried to establish growth charts to identify the risk of developing myopia in children,^36^ which concluded that the rate of eye growth was twice as high in the children who developed myopia compared to the children who remained hyperopic. Excessive elongation of the globe in adulthood is believed to contribute significantly to the degenerative changes of the retina that can lead to MMD and pathologic myopia, which can result in vision impairment in HM.^37^ Recent reports showed that severity of adolescent AL is associated with a thinner choroid, which is a known risk factor for MMD.^38^^,^^39^ However, more studies are necessary to ascertain the role of AL progression in predicting HM and the development of pathologic myopia and its associated complications.^40^

### Strengths and Limitations

A strength of this study is the longitudinal design with fairly long duration of follow-up; SE and AL were measured at eight different time points yearly with the majority of children having at least three or more follow-up visits. We were therefore able to assess the age of myopia onset and age of HM development, as well as progression rates of SE and AL. However, our study was limited by a loss to follow-up rate of 47%. Participants who attended the last follow-up visit were different compared to those who did not attend, and this may have introduced a selection bias. We also recognize the limitations of variability in A-scan biometry measurements introduced by factors, such as misalignment of beam and variable corneal compression, that may affect the accuracy of AL measurements. Although the combination of baseline SE and 3-year progression had the best AUC, knowing the SE progression over 3 years would be a potential impediment to its use in clinical practice. In situations where 3-year progression information is not available, progression over a shorter duration of time (1-year SE progression) could be considered, as it had a predictive value almost similar to that of 3-year progression, but 1-year progression may not be a stable parameter. In addition, the use of the more myopic eye (worse eye) in the analysis may be seen as a limitation as the clinician may not know at 3 years of follow-up which of the 2 eyes will be the more myopic at 6 to 8 years of follow-up. Nevertheless, the results from this study could serve as the precursor for studying myopia progression in childhood as a potential biomarker for HM and later pathologic myopia in adulthood. Ideally, final SE and AL in adulthood instead of the teenage years would provide more information. It is also important to note that multivariate ORs for SE and AL progression as well as baseline SE and AL showed wide 95% CI, which may be related to the sample size and number of events observed (only 91 children developed HM at the end of the follow-up). The estimated risk reported is also specific to the population studied and may be higher or lower in other populations. Thus, future studies, including larger sample size and longer follow-up until adulthood, may provide more precise estimates.

## Conclusion

In conclusion, our longitudinal cohort study with long follow-up suggests that 3 and 1-year myopia progression in childhood were predictors of teenage HM besides other key risk factors, including baseline SE and age of myopia onset. Teenagers with HM had greater SE progression in childhood, earlier onset of myopia and higher starting SE. A combination of 3 or 1-year myopia progression, baseline SE, and age of myopia onset may predict future HM. These combinations of factors could potentially guide clinical management, particularly in relation to myopia control treatment and counselling, to reduce the risk of developing HM and its associated ocular complications later on in life. Future studies with longer follow-up until adulthood would be helpful in ascertaining the effect of myopia progression in childhood on the risk of pathologic myopia development in adulthood.

## References

[1] Morgan IG, Ohno-Matsui K, Saw S-M. Myopia. *Lancet*. 2012; 379(9827): 1739–1748. 22559900 10.1016/S0140-6736(12)60272-4

[2] Morgan I, Rose K. How genetic is school myopia? *Prog Retin Eye Res*. 2005; 24(1): 1–38. 15555525 10.1016/j.preteyeres.2004.06.004

[3] Saw S-M, Matsumura S, Hoang Q V. Prevention and management of myopia and myopic pathology. *Investig Opthalmology Vis Sci*. 2019; 60(2): 488. 10.1167/iovs.18-25221 30707221

[4] Pan C-W, Ramamurthy D, Saw S-M. Worldwide prevalence and risk factors for myopia. *Ophthalmic Physiol Opt*. 2012; 32(1): 3–16. 22150586 10.1111/j.1475-1313.2011.00884.x

[5] Javitt JC, Chiang YP. The socioeconomic aspects of laser refractive surgery. *Arch Ophthalmol (Chicago, Ill 1960)*. 1994; 112(12): 1526–1530. 10.1001/archopht.1994.01090240032022 7993206

[6] Chen M, Wu A, Zhang L, et al. The increasing prevalence of myopia and high myopia among high school students in Fenghua City, eastern China: a 15-year population-based survey. *BMC Ophthalmol*. 2018; 18(1): 159. 29970057 10.1186/s12886-018-0829-8 PMC6029024

[7] Guo Y, Duan JL, Liu LJ, et al. High myopia in Greater Beijing School Children in 2016. Khanna RC, ed. *PLoS One*. 2017; 12(11): e0187396. 29121045 10.1371/journal.pone.0187396 PMC5679536

[8] Yotsukura E, Torii H, Inokuchi M, et al. Current prevalence of myopia and association of myopia with environmental factors among schoolchildren in Japan. *JAMA Ophthalmol*. 2019; 137(11): 1233–1239. 10.1001/jamaophthalmol.2019.3103 PMC6696729 31415060

[9] Jung S-K, Lee JH, Kakizaki H, Jee D. Prevalence of myopia and its association with body stature and educational level in 19-year-old male conscripts in Seoul, South Korea. *Investig Opthalmology Vis Sci*. 2012; 53(9): 5579. 10.1167/iovs.12-10106 22836765

[10] Inhoffen W, Ziemssen F. Morphologische Charakteristika der myopen choroidalen Neovaskularisation. *Der Ophthalmol*. 2012; 109(8): 749–757. 10.1007/s00347-011-2498-3 22911352

[11] Takeuchi K, Kachi S, Iwata E, Ishikawa K, Terasaki H. Visual function 5 years or more after macular translocation surgery for myopic choroidal neovascularisation and age-related macular degeneration. *Eye*. 2012; 26(1): 51–60. 22173070 10.1038/eye.2011.302 PMC3259604

[12] Coco Martín MB, Arranz De La Fuente I, González García MJ, Cuadrado Asensio R, Coco Martín RM. [Functional improvement after vision rehabilitation in low monocular vision after myopic macular degeneration and retinal detachment]. *Arch Soc Esp Oftalmol*. 2002; 77(2): 95–98. 11854861

[13] Rabb MF, Garoon I, LaFranco FP. Myopic macular degeneration. *Int Ophthalmol Clin*. 1981; 21(3): 51–69. 6169677 10.1097/00004397-198102130-00006

[14] Tideman JWL, Snabel MCC, Tedja MS, et al. Association of axial length with risk of uncorrectable visual impairment for Europeans with myopia. *JAMA Ophthalmol*. 2016; 134(12): 1355. 27768171 10.1001/jamaophthalmol.2016.4009

[15] Resnikoff S, Pascolini D, Mariotti SP, Pokharel GP. Global magnitude of visual impairment caused by uncorrected refractive errors in 2004. *Bull World Health Organ*. 2008; 86(1): 63–70. 18235892 10.2471/BLT.07.041210 PMC2647357

[16] Wong TY, Ferreira A, Hughes R, Carter G, Mitchell P. Epidemiology and disease burden of pathologic myopia and myopic choroidal neovascularization: an evidence-based systematic review. *Am J Ophthalmol*. 2014; 157(1): 9–25.e12. 24099276 10.1016/j.ajo.2013.08.010

[17] Neelam K, Cheung CMG, Ohno-Matsui K, Lai TYYY, Wong TY. Choroidal neovascularization in pathological myopia. *Prog Retin Eye Res*. 2012; 31(5): 495–525. 22569156 10.1016/j.preteyeres.2012.04.001

[18] Chua SYL, Sabanayagam C, Cheung Y-B, et al. Age of onset of myopia predicts risk of high myopia in later childhood in myopic Singapore children. *Ophthalmic Physiol Opt*. 2016; 36(4): 388–394. 27350183 10.1111/opo.12305

[19] Jensen H. Myopia in teenagers. *Acta Ophthalmol Scand*. 2009; 73(5): 389–393. 10.1111/j.1600-0420.1995.tb00294.x 8751114

[20] Hu Y, Ding X, Guo X, Chen Y, Zhang J, He M. Association of age at myopia onset with risk of high myopia in adulthood in a 12-year follow-up of a Chinese cohort [published online ahead of print September 17, 2020]. *JAMA Ophthalmol*, 10.1001/jamaophthalmol.2020.3451. PMC7499247 32940622

[21] Gwiazda J, Hyman L, Dong LM, et al. Factors associated with high myopia after 7 years of follow-up in the Correction of Myopia Evaluation Trial (COMET) Cohort. *Ophthalmic Epidemiol*. 14(4): 230–237. 17896302 10.1080/01658100701486459

[22] Saw S-M, Tong L, Chua W-H, et al. Incidence and progression of myopia in Singaporean school children. *Investig Opthalmology Vis Sci*. 2005; 46(1): 51. 10.1167/iovs.04-0565 15623754

[23] Saw S-M, Chua W-H, Hong C-Y, et al. Nearwork in early-onset myopia. *Invest Ophthalmol Vis Sci*. 2002; 43(2): 332–339. 11818374

[24] Saw S-M, Shankar A, Tan S-B, et al. A cohort study of incident myopia in Singaporean children. *Invest Ophthalmol Vis Sci*. 2006; 47(5): 1839–1844. 16638989 10.1167/iovs.05-1081

[25] Tong L, Carkeet A, Saw SM, Tan DTH. Corneal and refractive error astigmatism in Singaporean schoolchildren: a vector-based Javal's rule. *Optom Vis Sci*. 2001; 78(12): 881–887. 11780665 10.1097/00006324-200112000-00010

[26] Saw SM, Wu HM, Hong CY, Chua WH, Chia KS, Tan D. Myopia and night lighting in children in Singapore. *Br J Ophthalmol*. 2001; 85(5): 527–528. 11316706 10.1136/bjo.85.5.527 PMC1723973

[27] Saw S-M, Hong C-Y, Chia K-S, Stone RA, Tan D. Nearwork and myopia in young children. *Lancet*. 2001; 357(9253): 390. 10.1016/S0140-6736(05)71520-8 11211020

[28] Sacks D. Age limits and adolescents. *Paediatr Child Health*. 2003; 8(9): 577–577. 20019831 10.1093/pch/8.9.577 PMC2794325

[29] Saw SM, Chua WH, Hong CY, et al. Height and its relationship to refraction and biometry parameters in Singapore Chinese children. *Investig Ophthalmol Vis Sci*. 2002; 43(5): 1408–1413. 11980854

[30] Dirani M, Tong L, Gazzard G, et al. Outdoor activity and myopia in Singapore teenage children. *Br J Ophthalmol*. 2009; 93(8): 997–1000. 19211608 10.1136/bjo.2008.150979

[31] Jong Monica, He M, Holden BA, et al. The rate of myopia progression in children who become highly myopic. *Investig Opthalmology Vis Sci*. 2014; 13: 3636.

[32] Pärssinen O, Kauppinen M. Risk factors for high myopia: a 22-year follow-up study from childhood to adulthood. *Acta Ophthalmol*. 2019; 97(5): 510–518. 30460746 10.1111/aos.13964

[33] Ang M, Flanagan JL, Wong CW, et al. Review: Myopia control strategies recommendations from the 2018 WHO/IAPB/BHVI Meeting on Myopia. *Br J Ophthalmol*. 2020; 104(11): 1482–1487. 32102791 10.1136/bjophthalmol-2019-315575

[34] Chen Y, Zhang J, Morgan IG, He M. Identifying children at risk of high myopia using population centile curves of refraction. Pan C-W, ed. *PLoS One*. 2016; 11(12): e0167642. 28030593 10.1371/journal.pone.0167642 PMC5193395

[35] Tang SM, Kam KW, French AN, et al. Independent influence of parental myopia on childhood myopia in a dose-related manner in 2055 trios: the Hong Kong Children Eye Study. *Am J Ophthalmol*. 2020; 218: 199–207. 32454034 10.1016/j.ajo.2020.05.026

[36] Tideman JWL, Polling JR, Vingerling JR, et al. Axial length growth and the risk of developing myopia in European children. *Acta Ophthalmol*. 2018; 96(3): 301–309. 29265742 10.1111/aos.13603 PMC6002955

[37] Fricke TR, Jong M, Naidoo KS, et al. Global prevalence of visual impairment associated with myopic macular degeneration and temporal trends from 2000 through 2050: systematic review, meta-analysis and modelling. *Br J Ophthalmol*. 2018; 102(7): 855–862. 29699985 10.1136/bjophthalmol-2017-311266 PMC6047154

[38] Li J, Lanca C, Htoon HM, et al. High myopes in Singapore: 19-year progression from childhood to adulthood. *Ophthalmology*. 2020; 127(12): 1768–1770. 32445655 10.1016/j.ophtha.2020.05.031

[39] Devarajan K, Sim R, Chua J, et al. Optical coherence tomography angiography for the assessment of choroidal vasculature in high myopia. *Br J Ophthalmol*. 2020; 104(7): 917–923. 31585963 10.1136/bjophthalmol-2019-314769

[40] Wong CW, Foo LL, Morjaria P, et al. Highlights from the 2019 International Myopia Summit on “controversies in myopia” [published online ahead of print August 18, 2020]. *Br J Ophthalmol*, 10.1136/bjophthalmol-2020-316475. 32816799

